# A composition and size controllable approach for Au-Ag alloy nanoparticles

**DOI:** 10.1186/1556-276X-7-225

**Published:** 2012-04-18

**Authors:** Li Sun, Weiling Luan, Yue Jin Shan

**Affiliations:** 1 The Key Laboratory of Safety Science of Pressurized System (MOE), School of Mechanical and Power Engineering, East China University of Science and Technology, Shanghai, 200237, China; 2 Department of Applied Chemistry, Faculty of Engineering, Utsunomiya University, 7-1-2 Yoto, Utsunomiya, 321-8585, Japan

**Keywords:** Au-Ag alloy, Nanoparticles, Composition, Size, Micro-reaction method

## Abstract

A capillary micro-reaction was established for the synthesis of Au-Ag alloy nanoparticles (NPs) with a flexible and controllable composition and grain size by tuning the synthesis temperature, the residence time, or the mole ratio of Au^3+^:Ag^+^. By extending the residence time from 5 to 900 s, enhancing the temperature from 120°C to 160°C, or decreasing the mole ratio of Au^3+^:Ag^+^ from 1:1 to 1:20, the composition of samples was changed continuously from Au-rich to Ag-rich. The particles became large with the increase of the residence time; however, synthesis temperatures showed less effect on the particle size change. The particle size of the Au-Ag alloy NPs with various composition could be kept by adjusting the mole ratio of Au^3+^:Ag^+^. TEM observation displayed that the as-obtained NPs were sphere-like with the smallest average size of 4.0 nm, which is half of those obtained by the traditional flask method.

## Background

During the past decades, Au-Ag alloy has attracted great attentions due to its unique optical [1,2], electronic [3], and catalytic [4] properties. Especially, Au-Ag alloy displayed a comparable reduced reaction temperature when applied on CO oxidation in fields such as vehicle emissions, industrial waste, and the indoor atmosphere. Comparing with the traditional Au nanoparticles (NPs), Au-Ag alloy NPs showed a strong catalytic performance because of the synergistic effect of Au and Ag [5-8]. However, the synthesis of Au-Ag alloy NPs with mono-dispersed grain size, precise composition, and high surface activity is still a great challenge.

Several methods have been conducted on the synthesis of Au-Ag alloy, including laser ablation [9,10], phase-transfer [11], digestive ripening [12], co-reduction of Au and Ag salts [13,14], and galvanic replacement reaction [15]. Most of the reported methods relied on complex process, high cost, and strong ligands [13]. The co-reduction was regarded as a suitable method due to the Au-Ag NPs’ application preferability [13,16,17]. Wang et al. [13] found such products with the smallest size as 8 nm obtained in flask could achieve a complete conversion of CO at 150°C, while Au catalyst was previously tested inactive up to 300°C [18].

The micro-reaction system has some attractive advantages including high control accuracy, superior size and shape controllability, no inert atmosphere protection, and fast reaction rate [19]. Lin et al. [20] got Ag NPs with the smallest size of 5.6 nm by a continuous flow micro-reactor. Wagner and Köhler [21] and Köhler et al. [22-24] obtained Au NPs with the mean diameter between 24 nm and 35 nm in a chip micro-reactor. The size of the Ag and Au NPs was often tuned by changing the flow rate which was suitable for synthesizing mono-metal NPs, but not for bimetal NPs because of the variation of composition. Then, they synthesized star-like and sphere-like core-shell Au-Ag NPs with the average size between 80 nm and 120 nm in micro-fluidic systems by two or three steps; recently, a two-step micro-continuous flow-through method was used to synthesize multi-shell Au/Ag/Au NPs with the average size of 46.4 nm. However, Au-Ag alloy NPs with controlled size and composition are synthesized *via* micro-reaction, which has not been reported.

In this paper, the polytetrafluoroethylene (PTFE) capillary micro-reactor was adopted to synthesize Au-Ag alloy NPs where oleylamine (OLA) and octadecene (ODE) were taken as reductant and surfactant. The process parameters were systematically investigated. The composition and micro-structure of Au-Ag alloy NPs were deduced on the basis of the color of Au-Ag colloid. The structure, composition, and morphology were further studied by ultraviolet–visible spectrum (UV–vis), energy-dispersive spectrum (EDS), and transmission electron microscope (TEM).

## Methods

### Materials and set-up

Oleylamine (Aladdin Reagent Inc., Shanghai, People’s Republic of China), octadecene (Aladdin Reagent Company, Inc.), silver nitrate (AgNO_3_, Aladdin Reagent Inc.), chlorauric acid (HAuCl_4_·3H_2_O, Aladdin Reagent Inc.), ethanol (Aladdin Reagent Inc. and Sinopharm Chemical Reagent Co. Ltd., Shanghai, People’s Republic of China), and chloroform (Aladdin Reagent Inc. and Sinopharm Chemical Reagent Co. Ltd.) were used directly without further treatment. In our experiment, the capillary micro-reaction which was previously applied in the preparation of quantum dots was used to synthesize of Au-Ag NPs [25-28]. The syringe pump supplied a different flow rate which led to a variety of residence time. The blender and oil bath supported a stable temperature condition. The PTFE micro-reactor capillary (inside diameter: 750 μl; length: 100 cm), which is stable in the chemical reagents and can withstand moderate temperatures (up to 350°C), was coiled in oil bath. The structure of capillary enhanced the mass and heat transfer; moreover, it saved the space. Au-Ag colloid was collected in a collector and, then, separated by centrifuge (8,000 rpm, 10 min). The average size distribution of Au-Ag alloy was obtained by the statistical calculation of more than 150 particles from different parts of the grid.

### Characterization of the Au-Ag alloy NPs

EDS was applied to determine the mole ratio of Au and Ag using a scanning electron microscope (JSM-6360LV, JEOL, Tokyo, Japan) equipped with EDX (FALCON, EDAX, Kanagawa, Japan). Ultraviolet visible spectrum (UV–vis) analysis was carried out on a spectrophotometer (Cary 50, Varian Medical Systems, Inc., Tokyo, Japan). Chloroform was used as a standard for the background correction. The relationship between the composition of the Au-Ag NPs and absorption wavelength was investigated by UV–vis absorption spectra and EDS. A high-resolution transmission electron microscope (HR-TEM, JEM-2100 F, JEOL Ltd., Akishima, Tokyo, Japan) operated at 200 kV was used to observe the morphology. The sample for TEM was prepared by dipping an amorphous carbon-copper grid in a chloroform solution dispersed Au-Ag NPs homogeneously by sonicating for 5 min; then, the sample was left to evaporate in air at room temperature.

## Results and discussion

### Formation of Au-Ag alloy NPs

**Figure 1:**
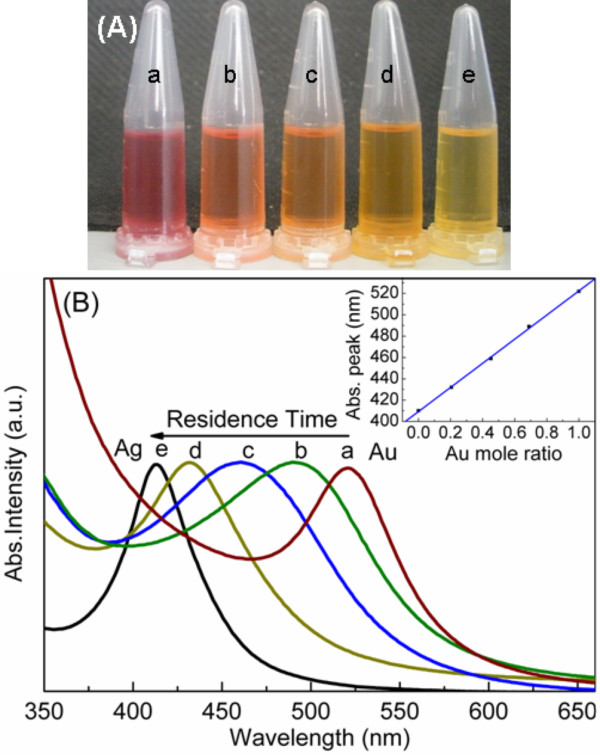
**The photograph of colloid (A), normalized UV–vis Abs. spectra (B).** Inset of (B) is the plot of the Abs. peak against the Au mole ratio of various Au-Ag alloy NPs. a: pure Au (abs, absorption: 522 nm); b-d: Au-Ag alloy (abs: 489, 459, and 432 nm, respectively) synthesized at 120°C with the Au^3+^:Ag^+^ ratio of 1:10; e: pure Ag (abs: 410 nm).

It was reported that the color of Au-Ag NPs colloid was a reflection of its composition and micro-structure [29,30]. Figure 1A shows the photographs of Ag, Ag-Au, and Au colloid prepared at 120°C. The color of Au colloid (a) is purple and the Ag colloid (e) is yellow, corresponding to their surface plasmons resonance absorption peak (Abs. peak) at 522 nm and 410 nm, respectively. The color of Au-Ag colloid (b, c, and d) gradually changes from purple to yellow with various Abs. peaks, which matched well with the color conversion rule of Au-Ag alloy colloid [31].

Figure 1B presents the normalized UV–vis absorption spectra of the samples. We can clearly find that all Au-Ag samples display only one Abs. peak which blue-shifted from 489 to 432 nm with the increase of the residence time from 120 to 640 s. For the physical mixture of Au and Ag NPs, there are two Abs. peaks between 522 and 410 nm [32]. When Au-Ag alloy was further added in, a third Abs. peak would show up, corresponding to a mixture of Au, Au-Ag alloy, and Ag [29]. Inset of the Figure 1B is the plot of the Abs. peaks against the Au mole ratio of various Au-Ag alloy NPs obtained from EDS, which manifested almost a linear relationship. It is commonly confirmed of a Au-Ag alloy formation when UV–vis absorption spectra show only one Abs. peak, and the Abs. peak against the mole ratios of Au exhibits a linear fashion [12,31,33].

### Composition control

**Figure 2:**
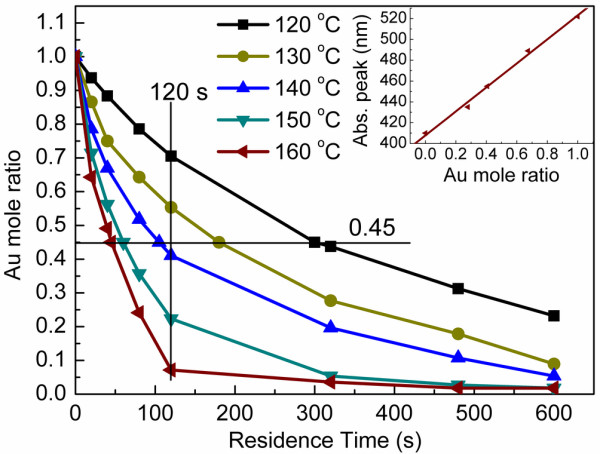
**Au mole ratio of samples synthesized under various residence times at different temperatures.** Ratio of Au mole while keeping the ratio of Au^3+^:Ag^+^ as 1:10. Inset is the plot of the Abs. peak against the Au mole ratio of various Au-Ag alloy NPs synthesized at 160°C. The ratio of Au^3+^:Ag^+^ is 1:10.

The composition of Au-Ag alloy can be tuned by either varying the residence time and temperature or adjusting the residence time and the mole ratio of Au^3+^:Ag^+^ in the raw material. Figure 2 denotes the Au mole ratio of Au-Ag alloy depended on various residence times and temperatures while keeping the mole ratio of Au^3+^:Ag^+^ as 1:10. The Au-Ag alloy NPs with the same composition can be synthesized at different temperature, and their composition from Au-rich to Ag-rich vary with increasing residence time. To obtain the same composition, it can be found that shorter residence time requires higher temperature. For example, the residence time to synthesize Au_0.45_Ag_0.55_ NPs is 300, 180, 105, 60, and 45 s, respectively, corresponding to the synthesis temperature changed from 120°C to 160°C. The residence time at 120°C is more than six times as that at 160°C. It can also be found that, at the same residence time, there is a Ag-rich trend with the increase of the synthesis temperature from 120°C to 160°C. Obviously, the reduction rate of Ag was enhanced at higher temperature. The faster reduction rate of Ag caused an Ag-rich alloy at the same residence time. Reaction temperature for Au^3+^ and Ag^+^ ions reduced to Au and Ag elements were reported as 65°C and 180°C, respectively [34,35]. When the synthesis temperature was lower than 180°C, the reduction rate of Ag^+^ was lower than that of Au^3+^. Besides, as the concentration of Ag^+^ was bigger than that of Au^3+^ (10:1), the increasing residence time resulted in the variation of composition from Au-rich to Ag-rich.

At higher temperature, the Au-Ag NPs may be a mixture of Au-Ag alloy, Ag or Au [13]. As shown in the inset of Figure 2, the plot of Abs. peak against the Au mole ratio of various Au-Ag alloy NPs displays an almost a linear relationship, which implies Au-Ag alloy can also be synthesized at 160°C.

**Figure 3:**
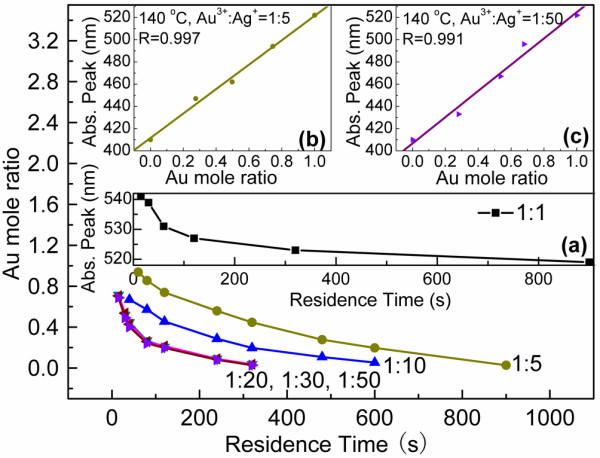
**Au mole ratio of samples synthesized under various residence times at different ratios of Au**^**3+**^**:Ag**^**+**^**.** Ratio of Au mole while keeping the temperature at 140°C. Inset (**a**) is the relationship between Abs. peak and the residence time. Inset (**b**) and (**c**) are the plots of the Abs. peak against the Au mole ratio of various Au-Ag alloy NPs. (a): 1:1, (b): 1:5, (c): 1:50.

Figure 3 exhibits the effect of mole ratios of Au^3+^:Ag^+^ on composition of samples synthesized under various residence times while keeping the temperature at 140°C. The Au-Ag alloy NPs with the same composition can be synthesized, and the Au mole ratio is decreased by increasing residence time at different mole ratios of Au^3+^:Ag^+^. It is hard to synthesize Au-Ag alloy when the ratio is 1:1 where the observed Abs. peak is bigger than Au (522 nm) in Figure 3 (a). At this condition, the obtained NPs were reported as core-shell structure [15,33,36]. Wang et al [13] reported a method to balance the reduction/growth processes between Au and Ag by decreasing the mole ratio of HAuCl_4_ to AgNO_3_. The higher concentration of AgNO_3_ could compensate its slower reduction rate and offer a comparative nucleation/growth speed with Au to form alloy. By adjusting the ratio to 1:5, the Au-Ag alloy from 515 nm to 413 nm has been synthesized. The residence time to obtain Au-Ag alloy with the same Au mole ratio is shortened by decreasing the mole ratio from 1:5 to 1:20, and there is no obvious change between 1:20 to 1:50. When the concentration of AgNO_3_ was high enough, the reductive amounts were decided by reducing agent. If the residence time is kept as a constant, it also can be found that the Au-Ag alloy with lower Au mole ratio can be obtained by decreasing the mole ratio of Au^3+^:Ag^+^.

It can be seen from Figure 3 insets (b) and (c) that the ideal linear fashions between the Abs. peak and the Au mole ratio of various Au-Ag alloy NPs are shown, which confirm the Au-Ag alloy NPs that were synthesized with the mole ratio changed from 1:5 to 1:50 at 140°C.

### Grain size control

**Figure 4:**
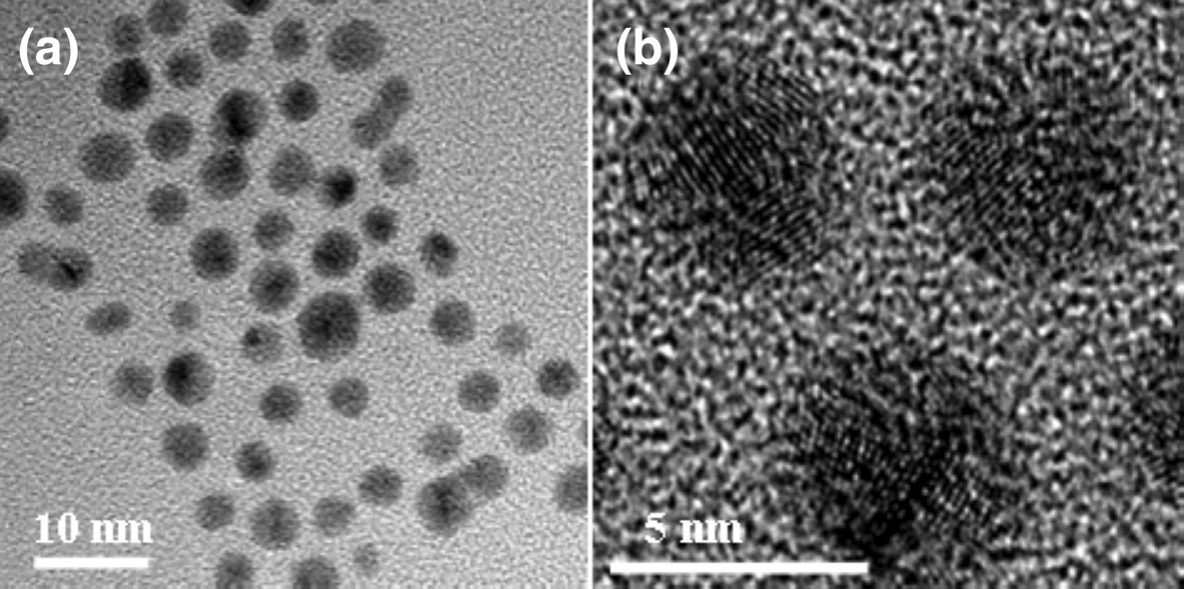
**Au mole ratio of samples synthesized under various residence times at different ratios of Au**^**3+**^**:Ag**^**+**^**.** Au-Ag alloy NPs with the Abs. peak of 509 nm were synthesized with the Au^3+^:Ag^+^ ratio of 1:10 at 120°C at 40 s.

Figure 4a represents the TEM image of Au-Ag alloy NPs with the Abs. peak of 509 nm, which clearly displays that the alloy NPs are sphere-like with the average size of 4 nm. As can be seen from Figure 4b, no evidence can be found to support that NPs have a core-shell structure, which further confirms that as-obtained Au-Ag NPs are of alloy structure. According to Wang’s report, the size of alloy NPs with the same Abs. peak as 509 nm, synthesized by the flask method, was 8.0 nm [13]. The fast transfer speed of capillary micro-reaction ensured a small average size of Au-Ag alloy NPs; however, Au-Ag alloy NPs still could not avoid the growth with the increasing residence time. When Au-Ag alloy NPs with the Abs. peak of 432 nm were synthesized at 640 s, their average size was near 7 nm. The same result was also got during the process of synthesizing the Au-Ag alloy by co-reduction of Au and Ag salts in aqueous solution method. It is well known that smaller size can cause a bigger surface and higher activity of NPs. However, it is hard to synthesize the Au-Ag alloy NPs with the same composition and size. Usually, the size of NPs was controlled *via* changing protective agents such as soluble polymers and organic ligands, or *via* the adsorption of anions on particle surface [37,38].

**Figure 5:**
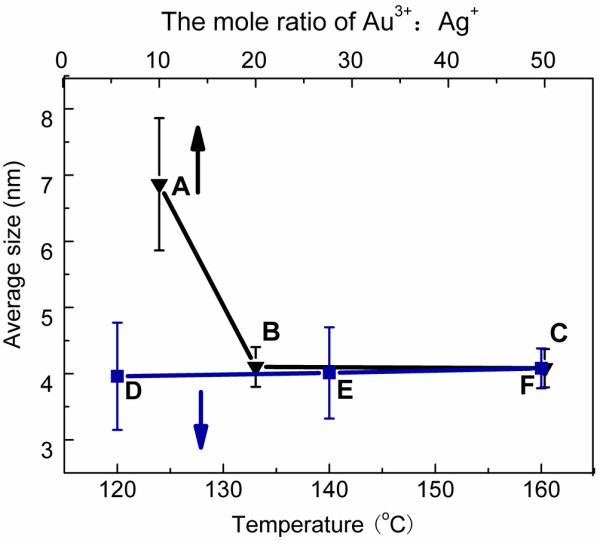
**The average size distribution of Au-Ag alloy NPs.** Average size distribution at different Abs. peak, temperature, residence time, and Au^3+^:Ag^+^ ratio. Sample A: 432 nm, 140°C, 320 s, 1:10; sample B: 432 nm, 140°C, 120 s, 1:20; sample C: 432 nm, 140°C, 120 s, 1:50; sample D: 509 nm, 120°C, 40 s, 1:10; sample E: 509 nm, 140°C, 15 s, 1:10; and sample F: 509 nm, 160°C, 8 s, 1:10. The black arrow means the **A, B, C** line uses the top axis. The blue arrow means the line **D, E, F** uses the bottom axis.

In our experiment, the size was tuned by changing the mole ratio of Au^3+^:Ag^+^. On the line of A, B, and C in Figure 5, the average sizes of Au-Ag alloy NPs with the same composition (432 nm) synthesized at different mole ratio of Au^3+^:Ag^+^ are exhibited. The average size of sample B is sharply lessened from 6.9 to 4.1 nm which is similar to sample E when the mole ratio is decreased from 1:10 to 1:20. After then, the size keeps constant with a value of about 4 nm. Comparing sample A with samples B and C, the residence time was shortened with the decrease of the mole ratio of Au^3+^:Ag^+^ from 320 to 120 s. The decreased residence time meant the growth time of Au-Ag alloy NPs was shortened which might lower the average size. Therefore, the size of Au-Ag alloy with different composition can also be kept stable by controlling the mole ratio of Au^3+^:Ag^+^. The method of tuning the size is helpful for synthesizing other alloys.

The synthesis temperature often has a strong effect on the size of NPs [39]. Figure 5 D, E, F line exhibits the relationship between average particle size of Au-Ag alloy NPs and synthesis temperature. Samples D, E, and F prepared at different temperatures (120°C, 140°C, and 160°C, respectively) during different residence times (40, 15, and 8 s, respectively) have the same composition (509 nm), and the average sizes are 4.0, 4.0, and 4.1 nm, respectively. Here, the little change of size indicates the Au-Ag alloy of the same composition, and size can be achieved at a shorter residence time by enhancing the temperature from 120°C to 160°C.

## Conclusions

The spherical Au-Ag alloy NPs with controllable composition and size were synthesized by a simple capillary micro-reaction method. The composition of Au-Ag alloy NPs could be tuned by controlling the synthesis conditions. The Abs. peak of Au-Ag alloy blue-shifted *via* enhancing the synthesis temperature, increasing the residence time, or decreasing the mole ratio of Au^3+^:Ag^+^. The Au-Ag alloy with the same composition and similar size could be obtained by enhancing the temperature from 120°C to 160°C, while the residence time got shorter. The Au-Ag alloy NPs changed from Au-rich to Ag-rich by increasing the residence time, corresponding with a growth of particle size. The size with different composition could be controlled as 4 nm by adjusting the mole ratio of Au^3+^:Ag^+^. Thus, micro-reaction provided a suitable and flexible way for producing composition and size-controlled Au-Ag alloys.

### Abbreviations

Abs, absorption; Abs. peak, absorption peak; EDS, energy-dispersive spectrum; HR-TEM, high-resolution transmission electron microscope; NPs, nanoparticles; ODE, octadecene; OLA, oleylamine; PTFE, polytetrafluoroethylene; TEM, transmission electron microscope; UV–vis, ultraviolet–visible spectrum.

## Competing interests

The authors declare that they have no competing interests.

## Authors’ contributions

The manuscript was written through the contributions of all authors. All authors read and approved the final manuscript.
